# Analysis of MDM2 and MDM4 Single Nucleotide Polymorphisms, mRNA Splicing and Protein Expression in Retinoblastoma

**DOI:** 10.1371/journal.pone.0042739

**Published:** 2012-08-20

**Authors:** Justina McEvoy, Anatoly Ulyanov, Rachel Brennan, Gang Wu, Stanley Pounds, Jinghui Zhang, Michael A. Dyer

**Affiliations:** 1 Department of Developmental Neurobiology, St. Jude Children's Research Hospital, Memphis, Tennessee, United States of America; 2 Department of Computational Biology, St. Jude Children's Research Hospital, Memphis, Tennessee, United States of America; 3 Department of Statistics, St. Jude Children's Research Hospital, Memphis, Tennessee, United States of America; 4 Department of Ophthalmology, University of Tennessee Health Science Center, Memphis, Tennessee, United States of America; 5 Howard Hughes Medical Institute, Chevy Chase, Maryland, United States of America; Yale Medical School, United States of America

## Abstract

Retinoblastoma is a childhood cancer of the developing retina that begins in utero and is diagnosed in the first years of life. Biallelic *RB1* gene inactivation is the initiating genetic lesion in retinoblastoma. The p53 gene is intact in human retinoblastoma but the pathway is believed to be suppressed by increased expression of MDM4 (MDMX) and MDM2. Here we quantify the expression of MDM4 and MDM2 mRNA and protein in human fetal retinae, primary retinoblastomas, retinoblastoma cell lines and several independent orthotopic retinoblastoma xenografts. We found that MDM4 is the major p53 antagonist expressed in retinoblastoma and in the developing human retina. We also discovered that MDM4 protein steady state levels are much higher in retinoblastoma than in human fetal retinae. This increase would not have been predicted based on the mRNA levels. We explored several possible post-transcriptional mechanisms that may contribute to the elevated levels of MDM4 protein. A proportion of MDM4 transcripts are alternatively spliced to produce protein products that are reported to be more stable and oncogenic. We also discovered that a microRNA predicted to target MDM4 (miR191) was downregulated in retinoblastoma relative to human fetal retinae and a subset of samples had somatic mutations that eliminated the miR-191 binding site in the MDM4 mRNA. Taken together, these data suggest that post-transcriptional mechanisms may contribute to stabilization of the MDM4 protein in retinoblastoma.

## Introduction

The p53 pathway is inactivated in virtually all human cancers [1]. Approximately half of human malignancies harbor mutations in the *TP53* gene itself while the remaining tumors with wild type *TP53* have genetic lesions in other key regulatory genes in the p53 pathway [2], [3]. For example, genetic amplification of the *MDM2* or *MDM4 (MDMX)* genes can lead to increased protein expression and suppression of the p53 response during tumorigenesis [3], [4], [5]. Moreover, recent data suggests that polymorphisms at the *MDM2* or *MDM4* loci may contribute to increased basal expression of these important p53 antagonists and increase cancer susceptibility [6], [7], [8].

Bond *et al.* identified two novel SNPs (SNP 309 and SNP 344) in the intronic promoter and first intron of *MDM2* [6]. SNP 309 T/G was present in 40% of the healthy volunteers in their cohort and SNP 309 G/G was found in 12% of the volunteers. SNP 344 was rare and was not analyzed further. Analysis of the sequence surrounding SNP 309 demonstrated that it was part of an SP1 consensus binding site. A series of biochemical and cellular analyses demonstrated that SNP309 G/G improved SP1 binding at the intronic promoter and increased MDM2 expression. Importantly, increased MDM2 expression led to partial suppression of the p53 pathway and a subsequent acceleration of tumorigenesis in Li Fraumeni patients [6].

In a similar study, *MDM4* SNPs were associated with breast and ovarian cancer risk [8]. In particular, SNP 7 T/T was found to associate with the early onset of familial and sporadic malignancies among individuals from families with elevated rates of breast and ovarian cancer [7]. Genotype data was collected for MDM4 SNP 7 among two independent cohorts of breast cancer patients (823 total patients). SNP7 T/T was associated with earlier age of onset for estrogen receptor negative breast cancers [7]. The underlying mechanism for the association of SNP7 T/T with earlier age of onset is not known. In a more recent study, Wynendaele and colleagues identified a SNP C>A in the 3′ UTR of MDM4 (SNP34091) that creates a putative target site for miR-191 [9]. The SNP34091-A allele is not efficiently recognized by miR-191 and this in turn leads to increased MDM4 protein expression and increased risk of high-grade carcinoma [9].

Retinoblastomas have wild type p53 [10], [11], [12] and cytogenetic studies have indicated that approximately 65% of retinoblastomas have genetic gain of *MDM4* [13], [14]. In a very small cohort of retinoblastoma samples, there was an association between *MDM4* gain and increased mRNA and protein expression [14]. However, the sample size was too small to provide statistical significance. Copy number alterations are rare in retinoblastoma ([10]) and MDM2 has not been analyzed for a relationship between genetic gain and gene or protein expression in retinoblastoma. We have recently confirmed that the p53 gene is wild type in a whole genome sequencing study of retinoblastoma [10] and these data are consistent with previously published data showing that retinoblastoma cells have an intact p53 response following DNA damage [14].

We recently performed gene expression array analysis of 52 human retinoblastomas and discovered that *MDM4* was expressed at high levels in all 52 tumors irrespective of the *MDM4* copy number [10], [15]. MDM2 was expressed at low levels in these 52 human retinoblastomas [15]. In a series of orthotopic xenografts of human retinoblastoma from our lab and Memorial Sloan Kettering Cancer Center (MSKCC) [16], MDM4 protein was expressed at high levels and MDM2 was below the limit of detection [15]. These data suggest that MDM4 expression may be elevated in retinoblastoma through mechanisms that are unrelated to the gene copy number. Specifically, MDM4 SNP7 T/T and/or SNP34901 A/A may contribute to tumor progression in retinoblastoma patients. It is also possible that MDM2 309 G/G contributes to tumorigenesis even though we could not detect the protein in human orthotopic xenografts. For example, MDM2 expression may be important for the initiation of retinoblastoma but it may be subsequently downregulated with concomitant upregulation of MDM4. Indeed, a recent study showed an association of the MDM2 309 G/G SNP with incidence of familial retinoblastoma [17]. There was no association with MDM4 SNP7 T/T in familial retinoblastoma in that study. Sporadic retinoblastoma has not been analyzed for MDM2 or MDM4 polymorphisms.

Here, we genotyped MDM4 SNP7, MDM4 SNP34091, and MDM2 SNP 309 in 44 retinoblastoma tumors, their corresponding blood DNA, and 3 human orthotopic xenografts. We compared the MDM4 and MDM2 SNP genotypes with gene expression and found no significant association. In human retinoblastoma orthotopic xenografts, we found no significant relationship between MDM4 SNP7 or MDM2 SNP 309 and their corresponding protein expression. However, all 3 of the orthotopic xenografts that we studied, had the MDM4 SNP34091 A/A allele and all had high levels of protein expression. Importantly, the level of MDM4 protein was elevated in retinoblastoma as compared to human fetal retina, but the mRNA levels were similar as measured with gene expression arrays and real time RT-PCR. These data suggest that MDM4 protein may be more stable and/or not subject to post-transcriptional repression in retinoblastoma as compared to human fetal retinae. The distribution of the MDM4 SNP34091 in our retinoblastoma cohort was not significantly different from that of the general population but we did identify somatic mutations C/A->A/A at SNP34091 in two patients. Moreover, miR-191 was significantly downregulated in retinoblastoma compared to human fetal retinae. Transcriptome analysis of 3 primary retinoblastoma samples revealed that a significant proportion of the MDM4 transcripts were alternatively spliced to produce the MDM4-S and MDM4-A forms of the protein that are believed to be oncogenic [18]. These transcript variants were validated by Sanger sequence in each of the orthotopic xenografts for the corresponding primary tumors. Taken together, these data suggest several possible mechanisms for elevated MDM4 protein expression, which are likely causal in suppressing the p53 pathway in retinoblastoma.

## Results

### Distribution of MDM2 SNP309 and MDM4 SNP7 in Human Retinoblastoma

We collected genomic DNA from tumor and blood samples from 44 human retinoblastoma patients, of which 32 were sporadic retinoblastoma cases, and 3 human orthotopic xenografts derived from independent primary tumors included in this cohort (SJ39-X, SJ41-X, and SJ42-X) (Table S1). The genomic region spanning MDM2 SNP309 and MDM4 SNP7 were PCR amplified and sequenced from both germline and tumor DNA samples as described previously [6], [8]. Due to poor sequence reads, 1–3 of the 44 samples could not be genotyped. The majority of MDM2 SNP309 (36/41 (88%)) and MDM4 SNP7 (39/43 (91%)) genotypes were identical between the tumor and germline samples (Table S2). Genotype sequence for all 3 xenograft tumors were identical to their respective primary tumor and germline samples (Table S2). For the discordant MDM2 SNP309 sequences, 4 were heterozygous in the germline (G/T) and homozygous in the tumor (T/T), which is the allele associated with lower expression of MDM2 [6]. Two were homozygous in the germline (T/T) and converted to the allele (G/G) allele associated with higher expression in the tumor (Table S2, SJRB048 and SJ07). For the discordant MDM4 SNP7 samples, 4 were heterozygous (T/C) in the germline sequence and homozygous (T/T or C/C) in the tumor sequence. Only one of these was found to contain the allele (T/T), which was previously reported to correlate with poor outcome in other types of cancer [8].

It is possible that these differences between the genotype of MDM2 SNP309 and MDM4 SNP7 are due to copy number changes of MDM2 or MDM4 in the tumor sample that result in overrepresentation of one allele in the tumor sample. To address this question, we performed SNP 6.0 analysis on each tumor and compared the data to the matched germline genomic DNA. We found that 3/4 of the samples with discordant SNP genotyping between germline and tumor had copy number gain of MDM4 in the tumor (Table S2, Materials and Methods S1). No perturbations were found at the MDM2 locus for those samples with discordant SNP genotypes between the tumor and the germline samples (Table S2).

### MDM2 SNP309 and MDM4 SNP7 genotypes do not correlate with gene expression in retinoblastoma

**Figure 1:**
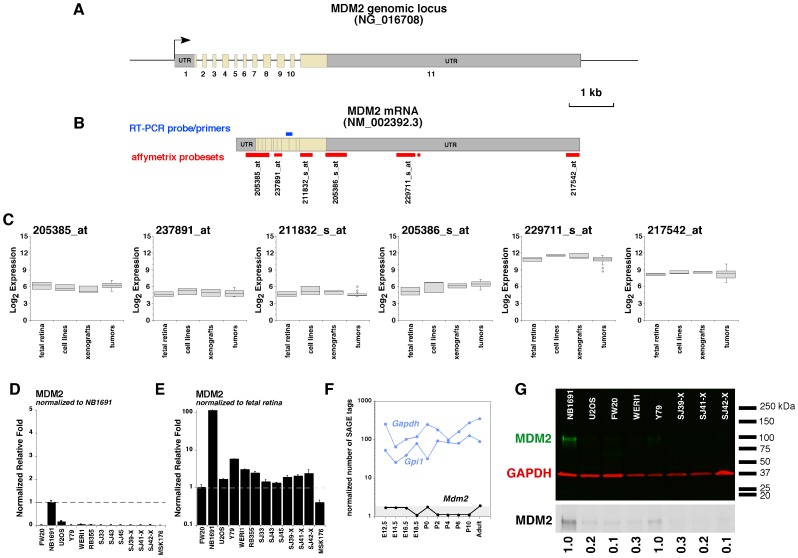
Expression of MDM2 in retinoblastoma. **A**) Structure of the genomic organization of the 11 exons of human *MDM2*. The exons are to scale as shown, but the introns are not to scale. **B**) Schematic of the spliced *MDM2* mRNA that produces the full-length MDM2 protein. The location and identification of Affymetrix gene expression array probesets are shown in red and the location of the real time RT-PCR probe/primer set is shown in blue. (**C**) Boxplots of the Log_2_ expression of each of the 6 probesets that uniquely mapped to *MDM2* for fetal retina, cell lines, xenografts and 52 primary human retinoblastomas. In general, genes expressed below Log_2_ of 7–8 cannot be detected by SAGE and are below the limit of reliable detection for retinal samples. (**D**) Real time RT-PCR for *MDM2* using Taqman probes as shown in (B) for fetal retina at gestational stage week 20 (FW20), a control (NB1691) that expresses high levels of MDM2, and a control (U2OS) that expresses high levels of MDM4, retinoblastoma cell lines (Y79, Weri1, RB355), primary tumors (SJ33, SJ43, SJ45), and retinoblastoma xenografts (SJ39-X, SJ41-X, SJ42-X and MSK176). Values were normalized to the positive control (NB1691). (**E**) The same data as shown in (D) but normalized to fetal retina. Each bar is the mean and standard deviation of duplicate experiments. (**F**) The SAGE data from developing mouse retina shows *Mdm2* is not expressed at significant levels in the developing mouse retina. The gray shaded box represents the limit of detection by SAGE (<2 normalized tags per sample). *Gapdh* and *Gpi1* are plotted as ubiquitiously expressed internal controls. (**G**) Immunoblot of MDM2 (green) protein in a subset of the samples analyzed by real time RT-PCR. Gapdh (red) was used as an internal reference for gel loading and the normalized values are presented below the black and white presentation of the blot for MDM2.

To test if there was any correlation between the SNP genotypes and MDM2 and MDM4 gene expression in retinoblastoma, we performed gene expression arrays on 22 of the 44 primary retinoblastoma tumors using the U133av_2 affymetrix chip. There are 9 probesets for MDM2, however 3 of the probesets (217373_x_at, 225160_x_at, and 244616_x_at) were not unique to MDM2 and were not analyzed further. The remaining 6 probesets were aligned to the human genome (NCBI 36/hg18) and mapped to the cDNA sequence for MDM2 (NM_002392.3). Three of the probesets shared homology with the 3′UTR of MDM2: one shared homology with exons 1–4, one shared homology with exons 6–7, and one shared homology with exon 11 (Figure 1A,B). In our previous experience with retinoblastoma gene expression array analysis, we have found that significant differences are virtually always validated by real time RT-PCR when the Log_2_>8.0 and this validation rate drops off dramatically below a Log_2_ value of 8.0 (McEvoy and Dyer, unpublished). Only probesets 229711_s_at and 215742_at showed levels of expression (Log_2_>8.0) in human fetal retinae and retinoblastoma (Figure 1C). There was no significant correlation between the MDM2SNP 309 genotype and the expression of any of the probesets (Table S3). Real time RT-PCR was consistent with this low level of MDM2 expression in fetal retinae, cell lines, primary tumors, and orthotopic xenografts (Figure 1D, E) and we could not detect MDM2 mRNA in the developing mouse retina from the retinal SAGE database [19] (Figure 1F). As a positive control for MDM2 expression, we used a cell line (NB1691) with amplification of MDM2 that leads to elevated MDM2 protein and suppression of p53 function (Figure 1D,E) [20]. We also used a cell line (U2OS) with an MDM4 amplification and no elevation of MDM2 as a control to provide a baseline of MDM2 expression in the context of the normal diploid gene copy number (Figure 1D,E) (C. Marine, personal communication). The expression of MDM2 was 10–100-fold higher in NB1691 than any of our retinoblastoma cell lines, orthotopic xenografts, primary tumor or human fetal retinae. Immunoblotting confirmed expression of full length MDM2 in NB1691 cells but there was very little MDM2 protein expressed in the retinoblastoma cell lines or xenografts except for the Y79 retinoblastoma cell line (Figure 1G and Figure S1).

**Figure 2:**
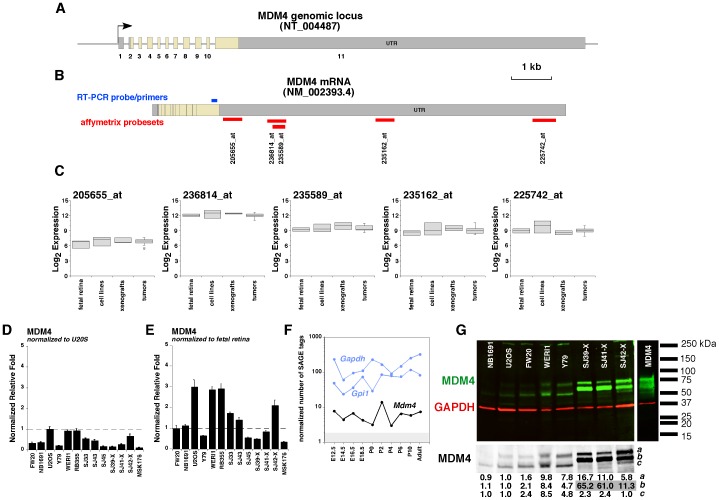
Expression of MDM4 in retinoblastoma. **A**) Structure of the genomic organization of the 11 exons of human *MDM4*. The exons are to scale as shown but the introns are not to scale. (**B**) Schematic of the spliced *MDM4* mRNA that produces the full-length MDM4 protein. The location and identification of Affymetrix gene expression array probesets are shown in red and the location of the real time RT-PCR probe/primer set is shown in blue. (**C**) Boxplots of the Log_2_ expression of each of the 5 probesets that uniquely mapped to *MDM4* for fetal retina, cell lines, xenografts and 52 primary human retinoblastomas. (**D**) Real time RT-PCR for MDM2 using Taqman probes as shown in (B) for fetal retina at gestational stage week 20 (FW20), control cell lines (NB1691 and U20S), retinoblastoma cell lines (Y79, Weri1, RB355), primary tumors (SJ33, SJ43, SJ45), and retinoblastoma xenografts (SJ39-X, SJ41-X, SJ42-X and MSK176). Values were normalized to the positive control (U2OS). (**E**) The same data as shown in (D) but normalized to fetal retina. Each bar is the mean and standard deviation of duplicate experiments. (**F**) The SAGE data from developing mouse retina shows *Mdm4* is expressed at significant levels in the developing mouse retina. The gray shaded box represents the limit of detection by SAGE (<2 normalized tags per sample). *Gapdh* and *Gpi1* are plotted as ubiquitiously expressed internal controls. (**G**) Immunoblot of MDM4 (green) protein in a subset of the samples analyzed by real time RT-PCR. Gapdh (red) was used as an internal reference for gel loading and the normalized values are presented below the black and white presentation of the blot for MDM4. The antibody was verified for specificity using a blocking peptide (not shown). Recombinant full length Flag-MDM4 protein was included as a positive control. Multiple bands were detected for MDM4 and were quantitated (below the black and white picture of the blot) and normalized to GAPDH and relative to U2OS cell line.

There are 7 probesets that recognize the MDM4 gene. One probeset (225740_x_at) was not unique to MDM4 and wasn't analyzed further. The remaining 6 probesets were aligned to the human genome (NCBI 36/hg18) and mapped to the cDNA sequence (NM_002393.4). One of the probesets (241876_at) did not align to MDM4 and was eliminated from our analysis. All the remaining 5 probesets shared homology with the 3′UTR of MDM4 found in all known transcript variants (Figure 2A,B and data not shown). 4 of the 5 probesets showed expression (Log_2_>8.0) across retinoblastoma samples (Figure 2C). We validated the gene expression data by real time RT-PCR using a Taqman probe to a unique region of exon 11 adjacent to the 3′UTR of MDM4 in fetal retinae, cell lines, primary tumors, and orthotopic xenografts (Figure 2B,D,E). Importantly, MDM4 is expressed at slightly higher levels than MDM2 in normal murine retinal development based on the retinal SAGE database [19] (Figure 2E, F). We also compared the expression of MDM4 mRNA to cell lines either previously shown to have MDM4 amplification (U2OS) as a control for MDM4 expression (Figure 2D,E) or MDM2 amplification (NB1691) as a control with normal levels of MDM4 (Figure 2D,E). Immunoblotting confirmed that MDM4 protein was expressed at high levels (∼5–65 fold increase) in our retinoblastoma orthotopic xenografts relative to fetal retina and U2OS cells (Figure 2G and Figure S1E). There was no correlation between MDM4 SNP7 genotype and gene expression for these samples for any of the probesets (Table S4). While the comparison of MDM2 and MDM4 protein levels across cell lines clearly shows that the relative abundance of the proteins is different, it is impossible to directly compare the absolute levels of MDM2 and MDM4 in retinoblastoma samples because the antibodies may have different affinities. Therefore, we performed a series of immunoblotting experiments using recombinant purified GST-MDM2 and GST-MDM4 protein to optimize the working antibody dilutions and to generate standard curves on the same blots as the tumor and cell line lysates (Figure S1, Materials and Methods S1). While the affinity of the MDM2 antibody used in this study was much lower than the MDM4 antibody, there was still significantly more MDM4 protein in retinoblastoma xenografts than MDM2 (>50 fold) (Figure S1).

### MDM4 has multiple transcript variants in retinoblastoma

**Figure 3:**
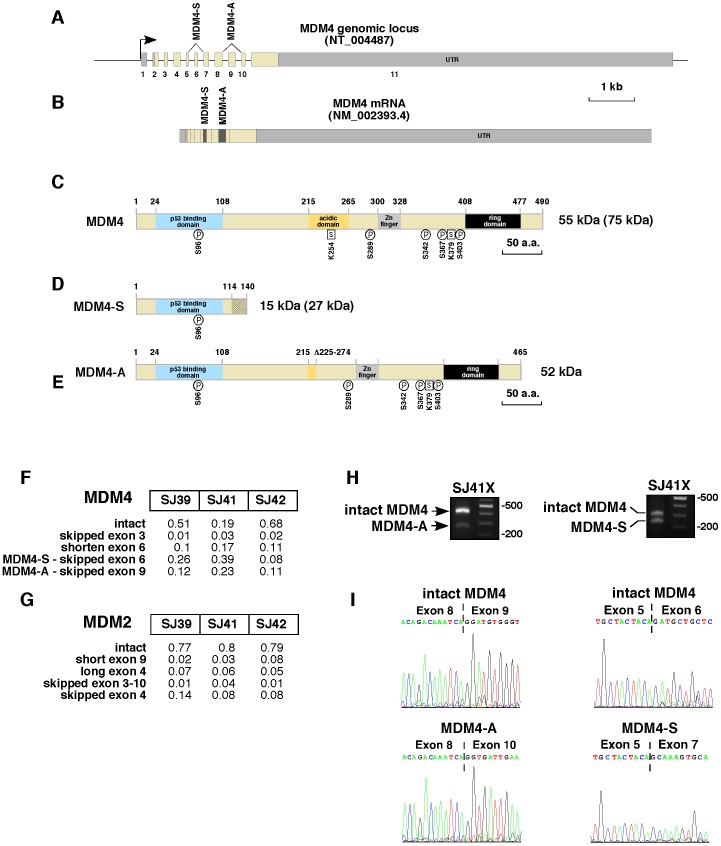
Analysis of transcript variants of MDM4 in retinoblastoma. **A, B**) Genomic organization of MDM4 showing two previously identified splice variants for this gene and the corresponding mRNA. MDM4-S results from skipping of exon 6 and MDM4-A results from skipping of exon 9 (dark gray shading). (**C**) Domain structure of the full length MDM4 protein with known protein modification sites (phosphorylation (P) and sumoylation (S)) ([18]). The predicted size is 55 kDa with an observed size on SDS-PAGE of ∼75 kDa. (**D**) The loss of exon 6 in MDM4-S results in a frameshift after residue 114 and a translational termination after residue 140 (diagonal line). The predicted size of MDM4-S is 15 kDa and the observed size on SDS-PAGE is 27 kDa [18], [27]. (**E**) The loss of exon 9 in MDM4-A results in an in frame fusion that is missing 50 residues spanning a portion of the acidic domain. (**F**) Representative reads from transcriptome analysis showing the loss of exon 6 and exon 9 in the retinoblastoma orthotopic xenografts. These alternative spliced transcripts were verified by PCR (**H**) and Sanger sequencing (**I**). (**G**) Representative reads from transcriptome analysis of MDM2 showing very little detection for alternative spliced transcripts.

Several different transcript variants have been reported for MDM4 (Reviewed in [18]) and these can result in proteins with altered mobility on immunoblots and altered stability *in vivo*. For example, the MDM4-S protein is the result of alternative splicing to remove exon 6 leading to a frameshift after residue 114 and premature termination of translation at residue 140 (Figure 3A, B, C). MDM4-S contains the p53 binding domain but lacks the downstream acidic and ring-finger domains (Figure 3D). The MDM4-S protein binds p53 with ∼10-fold greater affinity than full length MDM4 making it a potent oncogene [21], [22]. Similarly, the MDM4-A protein lacks exon 9 and is missing most of the acidic domain, but retains the p53 binding domain (Figure 3A,E) [23]. It has been hypothesized that the MDM4-A protein can act as an oncogene by enhancing the MDM2-mediated degradation of p53 [18]. To explore the MDM4 transcript variants in retinoblastoma, we performed transcriptome sequencing on 3 primary retinoblastomas that were used to generate the xenografts in this study (SJ39, SJ41, and SJ42). A proportion of the transcripts were altered to produce MDM4-S (8–39%) and MDM4-A (11–23%) (Figure 3F). In contrast to MDM4, the major MDM2 transcript present in SJ39, SJ41 and SJ42 was the full-length mRNA (Figure 3G). To validate the presence of the alternate MDM4 transcripts, we performed PCR and Sanger sequencing on orthotopic xenografts from the primary tumors (SJ39X, SJ41X and SJ42X) and cDNA from 3 independent primary tumors (Figure 3H,I and data not shown). We also identified MDM4-S and MDM4-A transcript variants in normal human fetal retinae (FW12, FW18, FW19 and FW20) and two independent adult retinae (data not shown).

### Analysis of the miR-191 binding site SNP in retinoblastoma

The transcriptome analysis showed that all of the transcripts expressed in the 3 retinoblastoma samples that were used to generate the orthotopic xenografts had the A/A allele of MDM4 SNP34091 which is the allele that is believed to be less sensitive to regulation by miR-191. To explore the possible role of miR-191 in retinoblastoma further, we genotyped SNP34091 in the tumor and normal DNA of each of samples in our cohort of 44 samples (Table S5). We found that 31/44 (70%) of the germline samples carried the A/A allele, 10/44 (23%) carried the C/A allele and 3/44 (7%) carried the C/C allele (Table S5). Interestingly, two germline samples with C/A alleles (SJ12 and SJ30) were converted to A/A alleles in the tumor by somatic mutation. We also genotyped all 3 orthotopic xenografts and found no sequence change from the primary tumor. Using the Wilcoxon rank-sum test, we found no correlation between the gene expression and MDM4 SNP34091 genotype (A/A and C/A) (Table S6). We only had one C/C sample in our cohort with gene expression data, so it was excluded from the statistical analysis. Also, statistical analysis showed there was no significant evidence of a biologically meaningful difference in genotype between retinoblastoma patients in our cohort and the HapMap cohort matched for race (p = 0.3926) (Table S7). When we performed Fisher's exact test for each race separately we found that there was a significant difference among blacks (p = 0.0254), driven largely by the absence of heterozygous genotypes among blacks retinoblastoma patients. However, allele frequencies among black patients and HapMap controls were very similar (Table S7).

**Figure 4:**
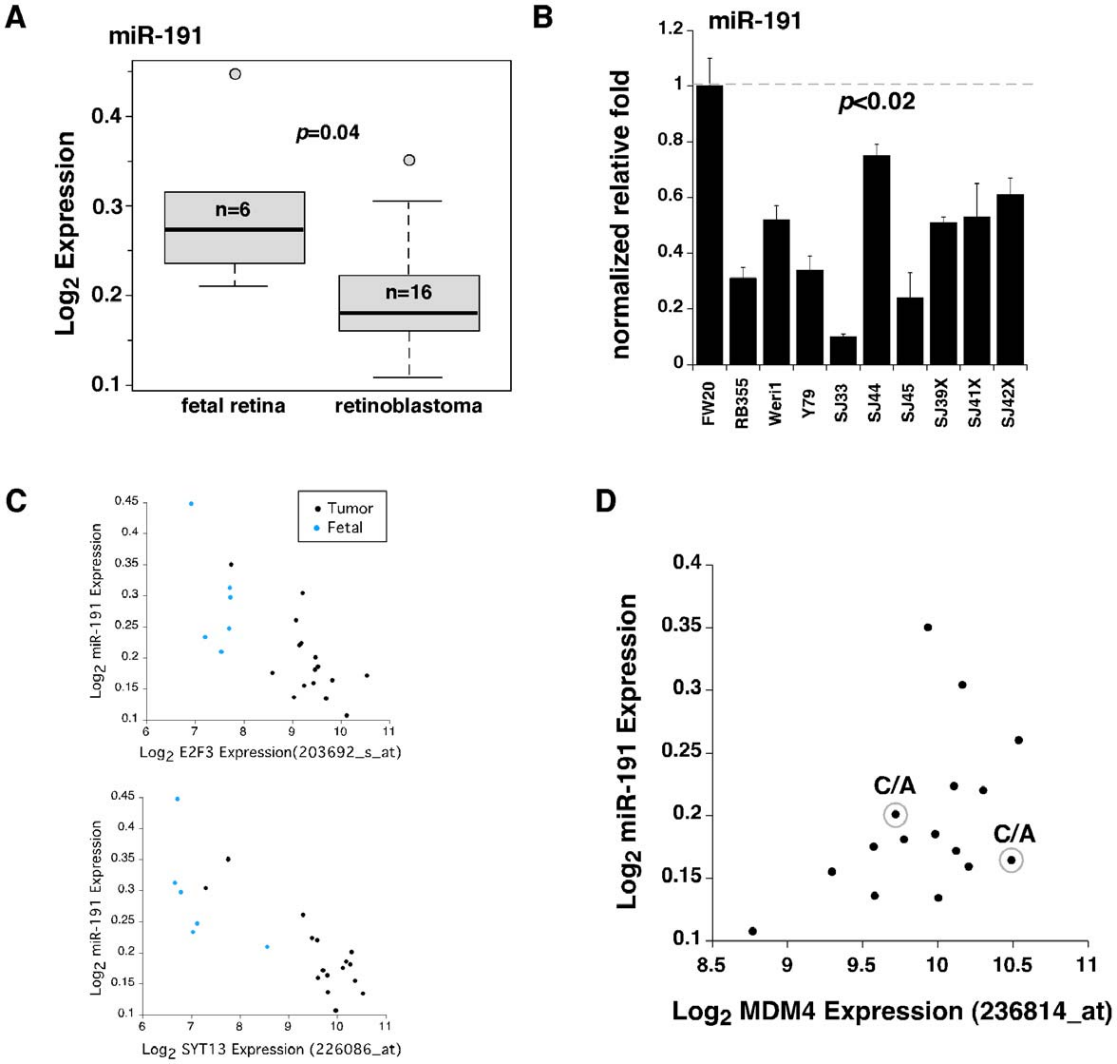
Analysis of miR-191 in retinoblastoma. **A**) Boxplot of the Log_2_ expression for hsa-mir-191 probeset in 6 fetal retina and 16 primary human retinoblastomas showing significantly lower expression. (**B**) MicroRNA real time-RT-PCR for hsa-mir-191 in fetal retina at gestational week 20, retinoblastoma cell lines (Rb355, Y79, and Weri1), retinoblastoma primary tumors, and orthotopic xenografts. Values are normalized to fetal retina. (**C**) Scatter plots of the Log_2_ expression values for hsa-mir-191 and 4 of the representative 44 mir-191 target genes in fetal retina and retinoblastoma primary tumors. Affymetrix probeset in parenthesis. (**D**) Scatter plot of the Log_2_ expression values for hsa-mir-191 and MDM4 (Affymetrix probeset 236814_at) in retinoblastoma primary tumors. Circled are primary tumors with a C/A genotype.

We analyzed the expression of miR-191, using the Agilent microRNA chip array in our human orthotopic xenografts, retinoblastoma cell lines, human fetal retinae and primary tumors. We found that miR-191 was significantly downregulated in retinoblastoma compared to human fetal retinae (p = 0.04) (Figure 4A). The miR-191 array data were validated by real time-RT-PCR (Figure 4B) and 19 target genes showed an inverse correlation between miR-191 levels and gene expression levels in our cohort (Figure 4C and Table S8). However, miR-191 expression levels did not significantly correlate with expression of MDM4 mRNA (r = 0.48; p = 0.097;) (Figure 4D). Additional samples with the C/A or C/C genotype would be required to provide statistically significant data on the relationship between miR-191 levels and MDM4 mRNA in retinoblastoma.

## Discussion

In this study, we analyzed the MDM2 and MDM4 mRNA and protein expression in human fetal retinae and human retinoblastoma. Our analysis included primary tumors, orthotopic xenografts and cell lines. We found that MDM4 is the major p53 antagonist expressed in retinoblastoma and the increased protein expression in the tumors compared to human fetal retinae would not have been predicted from the gene expression analysis. We propose that the increased MDM4 protein expression in retinoblastoma is regulated by post-transcriptional mechanisms. We demonstrated that the MDM4 mRNA is alternatively spliced in primary human retinoblastomas in a pattern that is predicted to produce altered versions of the MDM4 protein that have been shown to be oncogenic and more stable than the full-length protein. We also characterized the expression of miR-191 in retinoblastoma as compared to human fetal retinae and sequenced MDM4 SNP34091 where miR-191 binds. We found that the majority of primary retinoblastomas in our cohort (38/44) had at least one allele that is insensitive to miR-191. In addition, miR-191 was downregulated in retinoblastoma compared to human fetal retinae and multiple miR-191 targets were deregulated in retinoblastomas. Taken together, we propose that MDM4 protein levels are increased in retinoblastoma through multiple post-transcriptional mechanisms.

In our analysis, we found no correlation between the MDM2 SNP309 andMDM4 SNP7 genotype and mRNA levels. On average, the expression of MDM2 protein was 5-fold lower than the levels in a cell line (NB1691) with MDM2-mediated inactivation of p53 protein. The only exception was the Y79 retinoblastoma cell line. This cell line has drifted the furthest from primary retinoblastomas over the past 4 decades based on differences in gene expression array expression compared to primary retinoblastoma [15] so it is difficult to interpret the significance of this outlier. In contrast, the level of MDM4 protein in retinoblastoma cell lines and xenografts was up to 65 fold higher than human fetal retinae or a cell line (U2OS) that has MDM4 overexpression as a mechanism for inactivation of the p53 pathway. This observation was particularly interesting considering that the mRNA for MDM4 was not significantly elevated compared to human fetal retina. This observation led us to explore post-transcriptional mechanisms that may account for these elevated protein levels. We identified and validated two alternatively spliced forms of MDM4 in retinoblastoma orthotopic xenografts that have been previously reported. These transcripts produce different forms of the MDM4 protein (MDM4-A and MDM4-S) that are believed to be oncogenic and more stable than native MDM4 [18]. Interestingly, both of these forms of the MDM4 mRNA were also present in human fetal retinae raising the possibility that they play a role in normal development or predispose retinal cells to malignant transformation. A detailed study of the alternative forms of the MDM4 protein in the developing retina will be required to determine the significance of these alternative transcript variants in retinal development.

We also explored the role of miR-191 in regulating MDM4 mRNA and protein expression in retinoblastoma. It has been previously shown that MDM4 SNP34091 C allele creates a binding site for miR-191 and leads to reduced mRNA and protein levels [9]. In total, 41/44 retinoblastoma patients had at least one A allele for MDM4-SNP34091 and two of the patients with C/A alleles sustained somatic mutations in their tumors to create A/A alleles. However, we could not establish a statistically significant association between SNP34091 genotype and retinoblastoma susceptibility or MDM4 gene expression levels because of the relatively low proportion of MDM4 SNP34091 C/C allele in our cohort. These data have explored the multiple possible mechanisms for elevated MDM4 protein levels observed in retinoblastoma. Specifically, we propose that miR-191 expression levels, SNP34091 genotype and the relative abundance of MDM4-S and MDM4-A contribute to the steady state levels of MDM4 protein and the activity of the p53 pathway in retinoblastoma.

The MDM2 results derived from our study differ from those in the recent literature. Specifically, it was recently suggested that elevated levels of MDM2 protein was an important mechanism of p53 pathway suppression in retinoblastoma ([14], [16], [24], [25]). The data in support of this hypothesis was the observation that cone photoreceptors express MDM2 in the human retina and amacrine cells express MDM2 in the mouse retina [16]. Tumors derived from those species were reported to have features of cone and amacrine cells, respectively and it was inferred that the tumors must have arisen from the MDM2 expression cone and amacrine cells [16]. It has recently been confirmed that human retinoblastoma cells have features of photoreceptors and mouse retinoblastoma cells have features of amacrine cells [15]. However, human and mouse tumors express gene signatures of multiple cell types to produce a hybrid of photoreceptors, amacrine/horizontal interneurons and retinal progenitor cells. Indeed, the gene signature that was most consistent across species was the amacrine signature. Even at the level of individual cells, these normally incompatible gene expression signatures are co-expressed. Therefore, it is difficult to infer that the retinoblastoma tumor arose from MDM2 expressing cone photoreceptors in the human retina or MDM2 expressing amacrine cells in the mouse retina. More importantly, in that study, the investigators did not explore the expression of MDM4 and in our analysis [15] with the same xenografts used by Xu *et al.*, we show relatively high levels of MDM4 and undetectable levels of MDM2.

One intriguing possibility that we cannot exclude is that there are low levels of MDM2 protein that are below the limit of detection in our assays but may nonetheless contribute to tumorigenesis along with MDM4. We did detect some expression of MDM2 mRNA in our transcriptome analysis and gene expression array analysis and it is possible that MDM2 and MDM4 are working in concert to suppress the p53 pathway. It would be important to knock-down MDM2 in retinoblastoma orthotopic xenografts and study the p53 response in those cells *in vivo* to test this hypothesis. It is also possible that MDM2 and MDM4 work in sequence at different stages of tumorigenesis. For example, MDM2 may play a critical role in suppressing p53 during tumor initiation but then later it is downregulated and MDM4 expression is maintained at high levels in malignant retinoblastoma to suppress p53.

## Materials and Methods

### Ethics Statement

All experiments involving human subjects were approved by the St. Jude Children's Research Hospital Institutional Review Board and written/signed informed consent was obtained from the guardian on behalf of the minor (child) involved in the study, witnessed and filed in the chart of each patient.

The St. Jude Institutional Animal Care and Use Committee approved all animal procedures (IACUC protocol 393).

### Human Retinoblastoma Samples

Details for the acquisition of the tumor specimens used in this study have been preiously described [15]. For those samples with sufficient tissue, we performed SNP 6.0 analysis and gene expression arrays. For those samples with less tissue, we only performed SNP 6.0 analysis.

### Orthotpic Xenografts

Details for the retinoblastoma orthotopic xenografts used in this study have been previously described [15]. The MSKCC176 cells were kindly provided by Dr. David Cobrinik (Memorial Sloan-Kettering Cancer Center) [16].

### Cell Lines

Retinoblastoma cell lines Y79, Weri1 RB355, and NB1691 were cultured in RPMI medium (Lonza RPMI-1640) supplemented with 10% fetal bovine serum (Equitech Bio.), penicillin, streptomycin, and glutamate (Gibco). U2OS cell line was cultured in DMEM (Lonza DMEM 12-614F) supplemented with 10% fetal bovine serum (Equitech Bio.), penicillin, streptomycin, and glutamate (Gibco). Cells were passaged every 3 to 4 days or when they reached 70% to 80% confluence. Y79 and Weri1 cell lines were obtained from American Type Culture Collection (ATCC). The RB355 line was established in Brenda Gallie's lab (Ontario Cancer Institute, Toronto, Canada). We obtained NB1691 cell line from Jill Lahti at St. Jude Children's Research Hospital (SJCRH). This cell line was established by Peter Houghton (SJCRH) [26]. U2OS cell line was obtained from American Tissue Cell Culture (ATCC).

### DNA Extraction

5×10^6^ retinoblastoma cells were pelleted at 10,000× g for 1 min. and washed once in phosphate-buffered saline and pellet again. The cells were then resuspend in 480 µL DNA lysis buffer (10 mM Tris, 10 mM NaCl, 10 mM EDTA) with 12.5 µL 20% SDS and 10 µL proteinase K. The cells were lysed by incubating at 37°C overnight and then digested with 5 µL RNAse A (Qiagen) at 37°C for 10 min. We then added 10 µL 5 M NaCl and 1 µL 0.2 mg/mL glycogen and extracted with 500 µL buffer-saturated phenol. The genomic DNA was precipitated from the aqueous phase with ethanol and the pellet was washed with 70% ethanol before air drying for 10 minutes at room temperature. The DNA pellet was resuspended in 200- µL TE pH 8.0. DNA quality was assessed by spectrophotometry (Nanodrop).

### Sanger Sequencing

PCR was used to amplify DNA segments containing MDM4 SNP7 (rs1563828), MDM4 SNP34091 (rs4245739), and MDM2 SNP309 (rs2279744). Amplified PCR products were purified using PCR Purification Kit (Qiagen) and sequenced using Big Dye® Terminator (v3.1) Chemistry on Applied Biosystem 3730XL DNA Analyzers. The following primers were used for both the PCR amplification and Sanger sequencing: MDM2 SNP309 forward: 5′ CGGGAGTTCAGGGTAAAGGT 3′; MDM2 SNP309 reverse: 5′ AGCAAGTCGGTGCTTACCTG 3′; MDM4 SNP7 forward: 5′ GGGAAGGATCAACACCAGAAACAACC 3′; MDM4 SNP7 reverse: 5′ CAATCCCAAAGACAGACCCATAGGC 3′; MDM4 SNP34091 forward: 5′ ACGGGCCATCTTGTCACTTGTT 3′; MDM4 SNP34091 reverse: 5′ ACCTGACTGCTGCATAAAGTAATCCAT 3′.

### Gene Expression Arrays

Details for the gene expression arrays used in this study have been preiously described [15]. The gene expression array data were deposited in the GEO database (accession number: GSE29686).

### Correlation Analysis

Correlation analysis included genotypes for SNP7, SNP309, and SNP34091 and gene expression data for each patient sample. The Kruskal-Wallis test was used for three-group comparisons and the Wilcoxon rank-sum test was used for two-group comparisons between genotype and gene expression data.

### Real Time RT-PCR

Real-time RT-PCR experiments were performed using the Eppendorf Mastercycler ep Realplex2 system (Eppendorf, Germany). Primers and probes were designed using Primer Express® software (Applied Biosystems). TaqMan® probes were synthesized with 5′-FAM and 3′-BHQ. RNA was prepared using Trizol following manufacturer instructions (Invitrogen). cDNA was synthesized using the Superscript system (Invitrogen). Samples were analyzed in duplicate and normalized to Gapdh and Gpi1 expression levels. The following are the probes and primers used:

MDM2 forward: 5′ CTACAGGGACGCCATCGAAT 3′

MDM2 reverse: 5′ TGAATCCTGATCCAACCAATCA 3′

MDM2 probe: 5′ CGGATCTTGATGCTGGTGTAAGTGAACATTC 3′

MDM4 forward: 5′ TGGAAGGACGGGCCATCT 3′

MDM4 reverse: 5′ TGCTATAAAAACCTTAATAACCAGCTGAA 3′

MDM4 probe: 5′ TGTTTTCACTGTGCCAGAAGACTAAAGAAGG

### Immunoblotting

Total protein from cell lines Y79, Weri1, HEK293, NB1691, U2OS and 1–5 mg human xenograft retinoblastoma tissue was extracted with RIPA buffer (50 mM Tris-HCL pH8, 150 mM NaCl, 1% NP-40, 0.5% sodium Deoxycholate, 1% SDS, PMSF) with phosphatase inhibitors (Roche 11836153001). The concentration of lysates was determined by Bicinchoninic acid assay (Pierce 23225). 30 µg of lysate was electrophoresed on SDS-PAGE Tris-glycine gradient gels (4–20% BioRad 456-1093) and then transferred to nitrocellulose membranes. Membranes were blocked for 1 hour in Odyssey blocking buffer (LI-COR 927-40000) and then incubated with primary antibodies followed by IR Dye-labeled secondary antibodies diluted in Odyssey blocking buffer. Signals were measured using integrated intensity (counts) detected with an Odyssey infrared imaging system (LI-COR) at 680 and 800 nm. The following primary antibodies were used for immunoblotting: anti-mouse GAPDH 1∶5000 (abcam ab8245), anti-rabbit GAPDH 1∶5000 (abcam ab8485), anti-rabbit hMDMX/MDM4 1∶500 (Bethyl A300-287A-1), MDM2 SMP-14 mouse monoclonal IgG 1∶200 (Santa Cruz Biotechnology sc-965). The following secondary antibodies were used: anti-rabbit IgG (IRDye 680, LI-COR 926-32223), anti-goat IgG (IRDye 800, LI-COR 926-32214), and anti-mouse IgG (IRDye 800, LI-COR 926-32210).

### Cloning flag-tagged MDM4

Full-length MDM4 coding sequence was amplified using primers designed with a Not I restriction site encoded. The amplified PCR product was and cloned into pCR 2.1-TOPO (Invitrogen). Not 1 was used to excise the MDM4 cDNA from the pCR 2.1-TOPO vector and cloned into the Not1 sites of pCAG-GST (Addgene) vector downstream of 5′ flag tag. Expression of the construct was verified by western/immunoblotting using the monoclonal anti-flag 1∶2000 primary antibody (Sigma F3165-1 mg).

pCAG MDMX CDS For = 5′ GAGAGCGGCCGCAACATCATTTTCCACCTCTGCTCA 3′

pCAG MDMX CDS Rev = 5′ GAGAGCGGCCGCTGCTATAAAAACCTTAATAACCA 3′

### Transcriptome Sequencing and Analysis

RNA was purified from primary retinoblastoma (SJ39, SJ40, and SJ41) by phenol/chloroform/isoamyl alcohol (Invitrogen) extraction followed by sodium aetate/ethanol precipitation after each enzymatic step. For library construction 2–5 mg of total RNA was DNAse I (Invitrogen) treated for 15 minutes at 23°C, after which polyadenylated (poly-A) RNA was isolated with uMACS columns (Miltenyi Biotec). The isolated poly-A RNA served as a template for cDNA synthesis with random hexamers using the Superscript Double-Stranded cDNA Synthesis kit (Invitrogen). The resulting cDNA was fragmented by in a Covaris model E210 according to the manufacturer's recommended conditions to generate fragments with a peak distribution of approximately 200 bp. PAGE size selection was performed to further restrict the DNA size range to 100–300 bp prior to DNA end repair, and the addition of library adapters with the NEB Next DNA sample prep kit (NEB). After 10 rounds of PCR amplification with primers PE 1.0 and PE 2.0 (Illumina), the final product was size selected by PAGE (290–325 bp). The resulting libraries were quantitated with the QPCR NGS library quantification kit (Agilent Technologies) using a PhiX control library (Illumina) as an external standard. Bridge PCR clusters were generated on a V4 PE flow cell (Illumina) using a CBOT (Illumina). Flow cells were loaded onto an Illumina GAIIX for a paired end 2×101 cycle sequencing run using SCS version 2.8 software and SBS version 5 reagents, with the resulting base call files (bcl) converted to fastq format and used in the analysis pipeline. All Illumina paired-end reads were aligned to the following 4 database files using BWA (0.5.5) aligner: (1) human NCBI Build 36 reference sequence; (2) RefSeq; (3)Sequence file that represents all possible combination of non-sequential pairs of RefSeq exons; (4) AceView flatfile downloaded from UCSC which represents transcripts constructed from human EST. The mapping results from (2) to (4) were mapped to the human reference genome coordinates. The final BAM file was constructed by selecting the best alignment in the four databases. Coverage, SNV, indel and SV analysis were carried out by SJCRH using the methods described above.

### miRNA Arrays

#### Labeling

Cyanine-3 (Cy3) labeled miRNA was prepared from total RNA using the Agilent miRNA Microarray System with miRNA Complete Labeling and Hyb Kit, v.2.0 (June 2008, catalog #5190-0456) according to the manufacturer's instructions. Briefly, diluted spike-in mixture (catalog #5190-1934) and 100 ng of each total RNA was individually dephosphorylated using Calf Intestinal Phosphatase (CIP) for 30 minutes at 37°C, followed by denaturing in the presence of DMSO, at 100°C for 8 minutes. The samples were then snap cooled on ice for 5 minutes, followed by addition of Cy3-pCp and T4 RNA ligase, and incubation in the dark for 2 hours at 16°C. No post labeling purification was performed, followed by vacuum drying of the labeled reactions, per the protocol.

#### Hybridization

Per the protocol, the dried labeled products were re-suspended in nuclease-free water, followed by the addition of Agilent GE Blocking Agent and Agilent Hi-RPM Hybridization Buffer. The labeled miRNA was then heated to 100°C for 5 minutes, followed by 5 minutes in an ice-water bath. The entire volume (45 µl) of eight, individual reactions were hybridized to Agilent human miRNA 8×15K rel14 V2 G4471A-029297 microarrays, for 20 hours at 55°C, 20 rpm, in a rotating Agilent hybridization oven. After hybridization, microarrays were washed 5 minutes at room temperature with GE Wash Buffer 1 and 5 minutes with 37°C GE Wash Buffer 2 (Agilent GE wash buffer kit, catalog #5188-5327), then dried immediately by brief centrifugation.

#### Scanning

Slides were immediately scanned after washing on the Agilent Microarray Scanner (G2565CA) using one color scan setting for 8×15k array slides, using the following scan settings: 20-bit, scan area 61×21.6 mm, single pass, scan resolution 5 µm, 100% PMT, green dye channel only, and eXtended Dynamic range (XDR) not enabled.

### Mir-191 Target Gene Analysis

In order to find protein coding genes that are potentially regulated by miR-191, we downloaded the list of genes predicted to be targeted by miR-191 from several public databases (TargetScan: 34 genes; PicTar: 27 genes; MSKCC miRanda: 2,204 genes; Sanger microcosm: 2,208) and publications (Zhang et al.: 2 genes; Elyakim et al.: 39 genes) and merged into a non-redundant set (2,249 genes). We performed pair-wise correlation of the expression of miR-191 and that of its predicted targets. We selected significant genes with probesets if they had the correct inverse relationship between the miR sequence and the mRNA sequence and if they satisfied the following two criteria: 1) absolute correlation coefficient is >0.6; and 2) significantly differentially expressed between fetal and tumor samples (the false discovery rate adjusted p-value of student T-test<0.001.

### MicroRNA Real-Time RT-PCR

25 ng of RNA was used to make cDNA using the Taqman MicroRNA Reverse Transcription Kit (ABI PN 4366596) following manufacturer instructions. For quantitatve PCR, we used the TaqMan Small RNA assay and followed manufacturer instructions for hsa-mir-191 (ABI has-mir-191 assay ID # 002299) and endogenous control RNU6B (ABI 568915 assay ID # 001093 PN 4427975).

## Supporting Information

Figure S1 **MDM2 and MDM4 Antibody Optimization and Protein Quantification.** Recombinant purified GST-MDM2 1–185 and GST-MDM4 1–188 were used to optimize working dilutions for MDM2 (SMP-14) and MDM4 (A300-287A) antibodies. Two immunoblots of 5 10-fold serial dilutions of protein and 5 dilutions of antibody were performed to detect (A) GST-MDM2 (∼50 kDa) and (B) GST-MDM4 (∼50 kDa). (C–E) Protein was measured using Odyssey infrared imaging system (LI-COR) for two immunoblots with 2-fold serial dilutions of GST-MDM2 or GST-MDM4 alongside 30 µg of protein lysate from cell lines, fetal retina (gestational week 20), and human retinoblastoma orthotopic xenografts. The integrated intensity (counts) are plotted per nanograms of protein for (C) GST-MDM2 and (D) GST-MDM4 to generate a standard curve and linear trend line equation. (E) Integrated intensity (counts) measured for MDM2 and MDM4 in cell lines, fetal retina, and orthotopic xenografts were used to calculate the nanograms of protein based on the linear trend line equation from the standard curves (C, D). In table, a, b, and c refer to the different MDM4 bands as seen in figure 2G.
(TIF)

Table S1 **Clinical Features of Retinoblastoma Cohort.**
(PDF)

Table S2 **MDM2 SNP309 and MDM4 SNP 7 genotype in retinoblastoma patients.**
(PDF)

Table S3 **MDM2 expression levels for each MDM2 SNP309 genotype.**
(PDF)

Table S4 **MDM4 expression levels for each MDM4 SNP7 genotype.**
(PDF)

Table S5 **MDM4 SNP34091 genotype in retinoblastoma.**
(PDF)

Table S6 MDM4 expression levels for each MDM4 SNP34091 genotype.
(PDF)

Table S7 **SNP34091 Genotype Comparison Between Retinoblastoma Patients and HapMap Cohort.**
(PDF)

Table S8 **Target genes associated with mir-191 expression in retinoblastoma tumors.**
(PDF)

Materials and Methods S1 **Supplemental materials and methods and supporting references.**
(PDF)
