# Supplementary figures and images for: Analysis of MDM2 and MDM4 Single Nucleotide Polymorphisms, mRNA Splicing and Protein Expression in Retinoblastoma
Source: PLoS One. 2012 Aug 20;7(8):e42739. doi: 10.1371/journal.pone.0042739 (PMC3423419; doi:10.1371/journal.pone.0042739)

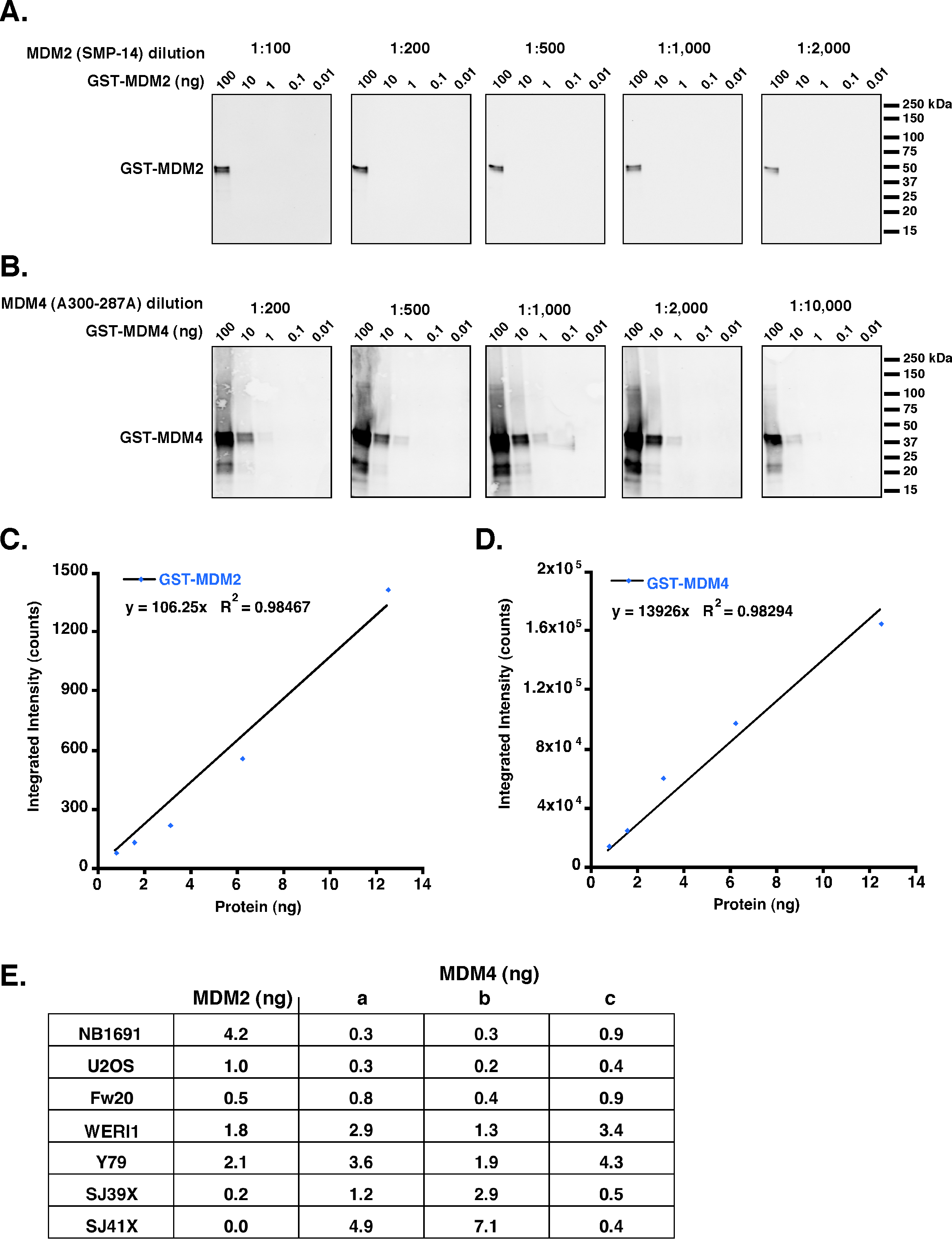

Supplement: Figure S1 — MDM2 and MDM4 Antibody Optimization and Protein Quantification. Recombinant purified GST-MDM2 1–185 and GST-MDM4 1–188 were used to optimize working dilutions for MDM2 (SMP-14) and MDM4 (A300-287A) antibodies. Two immunoblots of 5 10-fold serial dilutions of protein and 5 dilutions of antibody were performed to detect (A) GST-MDM2 (∼50 kDa) and (B) GST-MDM4 (∼50 kDa). (C–E) Protein was measured using Odyssey infrared imaging system (LI-COR) for two immunoblots with 2-fold serial dilutions of GST-MDM2 or GST-MDM4 alongside 30 µg of protein lysate from cell lines, fetal retina (gestational week 20), and human retinoblastoma orthotopic xenografts. The integrated intensity (counts) are plotted per nanograms of protein for (C) GST-MDM2 and (D) GST-MDM4 to generate a standard curve and linear trend line equation. (E) Integrated intensity (counts) measured for MDM2 and MDM4 in cell lines, fetal retina, and orthotopic xenografts were used to calculate the nanograms of protein based on the linear trend line equation from the standard curves (C, D). In table, a, b, and c refer to the different MDM4 bands as seen in figure 2G. (TIF) [file pone.0042739.s001.tif]
