# Supplementary material for: Analysis of MDM2 and MDM4 Single Nucleotide Polymorphisms, mRNA Splicing and Protein Expression in Retinoblastoma
Source: PLoS One. 2012 Aug 20;7(8):e42739. doi: 10.1371/journal.pone.0042739 (PMC3423419; doi:10.1371/journal.pone.0042739)
Supplement: Table S1 — Clinical Features of Retinoblastoma Cohort. (PDF) [file pone.0042739.s002.pdf]

**Supplemental Table 1. Clinical Features of Retinoblastoma Cohort**

| Sample | Sex | Ethnicity | Age (m) | [1]Stage | [2]Histology | [3]Growth | [4]Invasion | [5]Optic Nerve | Vitreal Seeds | RB1 mutation |
|---|---|---|---|---|---|---|---|---|---|---|
| SJ05 | M | black | 41 | Vb | FW, HW, nec | ? | Ch, Sc, AC, I | at | Y | Negative |
| SJ06 | F | white | 37 | Vb | undiff, fleur, nec | combo | N | N | Y | Negative |
| SJ07 | M | other | 11 | Va | FW, HW | ? | Ch | post | N | Positive |
| SJ12 | F | other | 67 | Vb | diff, HW, FW | ? | N | post | Y | Positive |
| SJ14 | M | other | 28 | Vb | diff, FW, nec | endo | Ch, SR | ant | Y | Negative |
| SJ16 | M | white | 45 | E, Vb | undiff | endo | N | N | Y | Negative |
| SJ17 | F | black | 37 | Vb | undiff | combo | Ch++, I, Sc | Post | Y | Negative |
| SJ18 | F | white | 7 | Va | undiff | exo | Ch | N | Y | Negative |
| SJ26 | M | black | 75 | Vb | diff, HW, FW | exo | N | ant | Y | Negative |
| SJ28 | M | black | 10 | D, Vb | undiff, FW, HW | combo | Ch | N | Y | Negative |
| SJ29 | M | other | 6 | Va | undiff/diff, FW, nec | exo | Ch++, SR | post | N | Negative |
| SJ30 | M | white | 33 | Vb | undiff, nec | endo | N | N | Y | Negative |
| SJ31 | M | black | 36 | Vb | diff | endo/combo | Ch | ant | Y | Negative |
| SJ32 | F | white | 32 | D, Va | undiff | exo | SR | ant | N | Negative |
| SJ33 | F | black | 13 | E, Vb | undiff, FW, HW | endo | Ch, SR | N | Y | Negative |
| SJ34 | M | unknown | 7 | E, Va | diff, HW, FW | exo | AC, SR | post | N | Negative |
| SJ35 | F | white | 3.5 | E, Vb | nec | combo | Ch++ | at | Y | Positive |
| SJ36 | F | white | 4 | ? | undiff, FW, HW | exo | Ch | ant | Y | Negative |
| SJ37 | F | white | 10 | Vb | undiff | exo | Ch, SR, CB | ant | Y | Positive |
| SJ38 | F | white | 23 | E, Va | undiff | exo | Ch++, Sc | at | N | Negative |
| SJ39 | M | white | 28 | E, Vb | undiff, nec | combo | Ch++ | post | Y | Negative |
| SJ40 | M | black | 13 | Vb | undiff, FW | exo | Ch, SR | ant | Y | Negative |
| SJ41 | F | white | 9 | Vb | undiff, FW | endo | Ch++ | Post | Y | Negative |
| SJ42 | F | white | 22 | Vb | undiff, FW | exo | SR | at | Y | Negative |
| SJ43 | M | black | 26 | E, Vb | undiff/diff, FW, HW | endo | SR | ant | Y | Negative |
| SJ44 | F | other | 17 | E, Vb | undiff, HW, FW | combo | N | ant | Y | Negative |
| SJ45 | F | other | 13 | E, Vb | undiff, FW, HW, nec | exo/combo | Ch, SR | ant | Y | Positive |
| SJ46 | M | white | 43 | E, Vb | undiff, nec | exo/combo | Ch, SR | ant | Y | Negative |
| SJ49 | F | white | 26 | D, Vb | undiff, fleur | endo | N | N | Y | Negative |
| SJ50 | M | white | 16 | Va | undiff, FW | diffuse | SR | N | N | Negative |
| SJRB005 | F | black | 22 | D, Vb | undiff, HW | combo | SR | at | Y | Negative |
| SJRB012 | F | black | 47 | D, Vb | Diff, FW | combo | Ch | N | Y | Negative |
| SJRB014 | M | white | 16 | ? | Diff | ? | N | N | Y | Negative |
| SJRB015 | F | black | 2 | ? | nec | combo | N | N | ? | Negative |
| SJRB016 | F | white | 3 days | ? | undiff | exo | Ch++, Sc | ant | N | Negative |
| SJRB028 | F | white | 17 | Vb | nec | ? | Ch, Sc | post | Y | Negative |
| SJRB029 | F | black | 26 | Vb | undiff, fleur | ? | AC, SR | ant | Y | Positive |
| SJRB030 | M | black | 10 | ? | diff, FW, nec | endo | Ch | ant | ? | Positive |
| SJRB031 | F | white | 77 | Vb | min nec | exo | AC, SR | post | Y | Negative |
| SJRB032 | M | white | 40 | ? | partial nec | ? | Ch | post | ? | Negative |
| SJRB033 | F | white | 22 | Vb | undiff | combo | Ch++ | post | Y | Positive |
| SJRB038 | M | white | 7 | Vb | undiff, FW, HW | exo | Ch | N | Y | Positive |
| SJRB047 | F | white | 9 | Vb | undiff+diff, nec | combo | SR | ant | Y | Positive |
| SJRB048 | M | other | 53 | ? | focal nec | ? | Ch | Post | ? | Not done |

[1] International retinoblastoma stage is listed first and Reese-Ellsworth stage is listed second.
[2] Retinoblastoma histology is classified as differentiated (diff), undifferentiated (undiff), necrotic (nec), fleurettes (fleur), Homer-Wright rosettes (HW) or Flexner-Wintersteiner rosettes (FW).
[3] Retinoblastoma growth is characterized as exophytic, endophytic or a combination of the two.
[4] Anterior chamber (AC), iris (I), ciliary body (CB), choroidal (Ch), massive choroidal (Ch++), subretinal (SR), scleral (Sc) invasion was scored for each sample.
[5] Optic nerve invasion scored as none (N), anterior (ant), at, or posterior (post) to lamina cribosa, or present at cut end of the optic nerve (end)
"?" indicates information is not available
