# Supplementary material for: Analysis of MDM2 and MDM4 Single Nucleotide Polymorphisms, mRNA Splicing and Protein Expression in Retinoblastoma
Source: PLoS One. 2012 Aug 20;7(8):e42739. doi: 10.1371/journal.pone.0042739 (PMC3423419; doi:10.1371/journal.pone.0042739)
Supplement: Table S2 — MDM2 SNP309 and MDM4 SNP 7 genotype in retinoblastoma patients. (PDF) [file pone.0042739.s003.pdf]

Supplemental Table 2. MDM2 SNP309 and MDM4 SNP 7 genotype in retinoblastoma patients

	MDM2 SNP309			MDM4 SNP7		
**Sample**	**Tumor**	**Germline**	**Copy number**	**Tumor**	**Germline**	**Copy number**
SJ05	T/T	T/T	0	T/T	T/T	1
SJ06	T/T	T/T	-1	C/C	C/C	0
SJ07**	T/T*	G/G*	0	T/C	T/C	0
SJ12**	T/T*	T/G*	0	T/C	T/C	0
SJ14	T/G	T/G	0	T/C	T/C	1
SJ16	T/T	T/T	0	T/C	T/C	1
SJ17	T/T	T/T	0	T/T	T/T	1
SJ18	T/G	T/G	0	T/T*	T/C*	1
SJ26	T/T	T/T	0	C/C	C/C	0
SJ28	T/T	T/T	0	C/C	C/C	0
SJ29	T/T*	T/G*	0	C/C	C/C	0
SJ30	T/G	T/G	0	C/C*	T/C*	1
SJ31	T/T	T/T	0	T/C	T/C	1
SJ32	T/G	T/G	0	C/C	C/C	1
SJ33	N/A	N/A	0	T/T	T/T	0
SJ34	T/T	T/T	0	C/C	C/C	1
SJ35**	T/T*	T/G*	0	T/C	T/C	0
SJ36	T/T	T/T	0	C/C	C/C	0
SJ37**	G/G	G/G	0	C/C*	T/C*	1
SJ38**	N/A	N/A	0	C/C	C/C	1
SJ39	T/G	T/G	0	C/C	C/C	0
SJ39-X	T/G	T/G	0	C/C	C/C	0
SJ40	T/T	T/T	0	C/C	C/C	0
SJ41**	T/G	T/G	0	C/C	C/C	0
SJ41-X**	T/G	T/G	0	C/C	C/C	0
SJ42	G/G	G/G	0	C/C	C/C	1
SJ42-X	G/G	G/G	0	C/C	C/C	1
SJ43	T/T	T/T	0	C/C	C/C	1
SJ44	T/G	T/G	0	C/C*	T/C*	1
SJ45**	G/G	G/G	0	C/C	C/C	1
SJ46	T/T	T/T	0	T/C	T/C	1
SJ49	T/T	T/T	-1	C/C	C/C	1
SJ50	T/T	T/T	0	T/C	T/C	0
SJRB005	T/T	T/T	0	T/C	T/C	1
SJRB012	T/T	T/T	-1	T/T	T/T	1
SJRB014	T/G	T/G	0	T/C	T/C	1
SJRB015	T/G	T/G	0	N/A	N/A	0
SJRB016	T/T	T/T	0	C/C	C/C	0
SJRB028	T/T*	T/G*	0	C/C	C/C	0
SJRB029**	T/T	T/T	0	T/T	T/T	1
SJRB030**	T/T	T/T	0	T/C	T/C	0
SJRB031	T/G	T/G	0	C/C	C/C	0
SJRB032	T/T	T/T	0	C/C	C/C	1
SJRB033**	T/G	T/G	0	T/C	T/C	1
SJRB038**	N/A	N/A	0	C/C	C/C	0
SJRB047**	G/G	G/G	0	C/C	C/C	0
SJRB048	T/T*	G/G*	-1	C/C	C/C	1

X indicates orthotopic xenograft sample

N/A indicates sequence is not available for this sample.

"1" indicates a gain in one copy of the MDM2 or MDM4 locus changing the total copy number to 3.

"-1" indicates a loss in one copy of the MDM2 or MDM4 locus changing the total copy number to 1.

"0" indicates no change in the diploid state for the MDM2 or MDM4 locus.

* Discordant genotype

** Rb1 germline mutation
