# Supplementary material for: Analysis of MDM2 and MDM4 Single Nucleotide Polymorphisms, mRNA Splicing and Protein Expression in Retinoblastoma
Source: PLoS One. 2012 Aug 20;7(8):e42739. doi: 10.1371/journal.pone.0042739 (PMC3423419; doi:10.1371/journal.pone.0042739)
Supplement: Table S3 — MDM2 expression levels for each MDM2 SNP309 genotype. (PDF) [file pone.0042739.s004.pdf]

Supplemental Table 3. MDM2 expression levels for each MDM2 SNP309 genotype.

		205385_at		*205386_s_at*		*211832_s_at*		*217542_at*		*229711_s_at*		*237891_at*	
**Genotype**	**# of samples**	**Mean**	**Std. Dev.**	**Mean**	**Std. Dev.**	**Mean**	**Std. Dev.**	**Mean**	**Std. Dev.**	**Mean**	**Std. Dev.**	**Mean**	**Std. Dev.**
T/T	13	5.95	0.6	6.46	0.54	4.96	0.52	8.08	0.86	10.68	0.68	5.11	0.59
T/G	5	6.3	0.63	6.6	0.52	4.73	0.28	8.95	0.7	10.73	0.36	5.39	0.56
G/G	3	5.99	0.32	6.39	0.69	4.82	0.05	8.69	0.04	10.33	1.47	4.85	0.49
		p value = 0.4975		*p value = 0.8681*		*p value = 0.4629*		*p value = 0.0767*		*p value = 0.9183*		*p value = 0.5814*	

The *p value* reflects the statistical significance of the correlation between SNP genotypes and gene expression.
