# Supplementary material for: Analysis of MDM2 and MDM4 Single Nucleotide Polymorphisms, mRNA Splicing and Protein Expression in Retinoblastoma
Source: PLoS One. 2012 Aug 20;7(8):e42739. doi: 10.1371/journal.pone.0042739 (PMC3423419; doi:10.1371/journal.pone.0042739)
Supplement: Table S4 — MDM4 expression levels for each MDM4 SNP7 genotype. (PDF) [file pone.0042739.s005.pdf]

Supplemental Table 4. MDM4 expression levels for each MDM4 SNP7 genotype.

		205655_at		*236814_at*		*235589_at*		*235162_at*		*225742_at*	
**Genotype**	**# of samples**	**Mean**	**Std. Dev.**	**Mean**	**Std. Dev.**	**Mean**	**Std. Dev.**	**Mean**	**Std. Dev.**	**Mean**	**Std. Dev.**
C/C	14	6.746813	0.65009	11.90514	0.373864	11.04347	0.561138	9.079377	0.640434	8.9798007	0.49757
T/C	5	7.16846	0.338023	12.0092	0.38993	11.14278	0.481391	8.53062	0.366049	8.8537875	0.534494
T/T	3	7.091993	0.336163	11.75437	0.966777	11.09357	0.714262	9.23664	0.290161	9.4168267	0.62526
		p value = 0.663		*p value = 0.2952*		*p value = 0.4833*		*p value = 0.0668*		*p value = 0.1925*	

The *p value* reflects the statistical significance of the correlation between SNP genotypes and gene expression.
