# Supplementary material for: Analysis of MDM2 and MDM4 Single Nucleotide Polymorphisms, mRNA Splicing and Protein Expression in Retinoblastoma
Source: PLoS One. 2012 Aug 20;7(8):e42739. doi: 10.1371/journal.pone.0042739 (PMC3423419; doi:10.1371/journal.pone.0042739)
Supplement: Table S5 — MDM4 SNP34091 genotype in retinoblastoma. (PDF) [file pone.0042739.s006.pdf]

Supplemental Table 5. MDM4 SNP34091 genotype in retinoblastoma

	MDM4 SNP34091		
Sample	Tumor	Germline	Copy number
SJ05	C/C	C/C	1
SJ06	A/A	A/A	0
SJ07**	C/A	C/A	0
SJ12**	A/A*	C/A*	0
SJ14	A/A	A/A	1
SJ16	C/A	C/A	1
SJ17	A/A	A/A	1
SJ18	A/A	A/A	1
SJ26	A/A	A/A	0
SJ28	A/A	A/A	0
SJ29	A/A	A/A	0
SJ30	A/A*	C/A*	1
SJ31	A/A	A/A	1
SJ32	C/C*	A/A*	1
SJ33	N/A	A/A	0
SJ34	A/A	A/A	1
SJ35**	C/A	C/A	0
SJ36	A/A	A/A	0
SJ37**	C/A	C/A	1
SJ38**	A/A	A/A	1
SJ39	A/A	A/A	0
SJ39-X	A/A	A/A	0
SJ40	A/A	A/A	0
SJ41**	A/A	A/A	0
SJ41-X**	A/A	A/A	0
SJ42	A/A	A/A	1
SJ42-X	A/A	A/A	1
SJ43	A/A	A/A	1
SJ44	C/A	C/A	1
SJ45**	A/A	A/A	1
SJ46	C/A	C/A	1
SJ49	A/A	A/A	1
SJ50	C/A	C/A	0
SJRB005	A/A	A/A	1
SJRB012	A/A	A/A	1
SJRB014	C/A	C/A	1
SJRB015	C/C	C/C	0
SJRB016	A/A	A/A	0
SJRB028	C/C*	A/A*	0
SJRB029**	C/C	C/C	1
SJRB030**	A/A	A/A	0
SJRB031	A/A	A/A	0
SJRB032	A/A	A/A	1
SJRB033**	A/A	A/A	1
SJRB038**	A/A	A/A	0
SJRB047**	A/A	A/A	0
SJRB048	A/A	A/A	1

X indicates orthotopic xenograft sample
N/A indicates sequence is not available for this sample.
"1" indicates a gain in one copy of the MDM4 locus changing the total copy number to 3.
"0" indicates no change in the diploid state for the MDM4 locus.
* Discordant genotype
** Rb1 germline mutation
