# Supplementary material for: Analysis of MDM2 and MDM4 Single Nucleotide Polymorphisms, mRNA Splicing and Protein Expression in Retinoblastoma
Source: PLoS One. 2012 Aug 20;7(8):e42739. doi: 10.1371/journal.pone.0042739 (PMC3423419; doi:10.1371/journal.pone.0042739)
Supplement: Table S6 — MDM4 expression levels for each MDM4 SNP34091 genotype. (PDF) [file pone.0042739.s007.pdf]

Supplemental Table 6. MDM4 expression levels for each MDM4 SNP34091 genotype.

		205655_at		***225742_at***		***235162_at***		***236814_at***		***235589_s_at***	
**Genotype**	**# of samples**	**Mean**	**Std. Dev.**	**Mean**	**Std. Dev.**	**Mean**	**Std. Dev.**	**Mean**	**Std. Dev.**	**Mean**	**Std. Dev.**
A/A	17	6.82	0.62	8.98	0.47	9.03	0.62	11.91	0.50	11.05	0.56
C/A	4	6.91	0.63	8.96	0.54	8.69	0.39	11.83	0.20	10.93	0.36
		p value = 1		*p value = 0.9654*		*p value = 0.275*		*p value = 0.4104*		*p value = 0.6977*	

The *p value* reflects the statistical significance of the correlation between SNP genotypes and gene expression.
