# Supplementary material for: Analysis of MDM2 and MDM4 Single Nucleotide Polymorphisms, mRNA Splicing and Protein Expression in Retinoblastoma
Source: PLoS One. 2012 Aug 20;7(8):e42739. doi: 10.1371/journal.pone.0042739 (PMC3423419; doi:10.1371/journal.pone.0042739)
Supplement: Table S7 — SNP34091 Genotype Comparison Between Retinoblastoma Patients and HapMap Cohort. (PDF) [file pone.0042739.s008.pdf]

Supplemental Table 7: SNP34091 Genotype Comparison Between Retinoblastoma Patients and HapMap Cohort

	*Black**		*White**	
**Genotype**	**Patients**	**HapMap**	**Patients**	**HapMap**
AA	9	31	14	36
AC	0	19	6	15
CC	3	6	0	3
	p value = 0.0254		*p value* = 0.8047	

The Mantel-Haenzel test was used to compare genotype frequencies of RB cases to that of HapMap among both whites and blacks while stratifying for race. This test found no significant association (p = 0.3926).

*Subjects of other or mixed racial backgrounds were excluded due to small sample size in our retinoblastoma cohort.
