# Supplementary material for: Analysis of MDM2 and MDM4 Single Nucleotide Polymorphisms, mRNA Splicing and Protein Expression in Retinoblastoma
Source: PLoS One. 2012 Aug 20;7(8):e42739. doi: 10.1371/journal.pone.0042739 (PMC3423419; doi:10.1371/journal.pone.0042739)
Supplement: Table S8 — Target genes associated with mir-191 expression in retinoblastoma tumors. (PDF) [file pone.0042739.s009.pdf]

Supplemental Table 8. Target genes associated with mir-191 expression in retinoblastoma tumors.

Gene	Probeset	corr	p.fdr
SYT13	226086_at	-0.808	0.00033
ATP1B1	201242_s_at	-0.762	2.27E-05
ATP1B1	201243_s_at	-0.756	2.05E-06
LOC149134	230541_at	-0.740	0.00045
E2F3	203692_s_at	-0.723	2.87E-06
SLC12A2	204404_at	-0.673	2.76E-07
RABGAP1L	215342_s_at	-0.669	2.48E-06
PHLDA2	209803_s_at	-0.665	5.63E-05
CEP164	1558953_s_at	-0.659	0.00072
SLC39A14	212110_at	-0.654	0.0007
GREM2	220794_at	-0.648	2.23E-05
GNG4	205184_at	-0.646	1.93E-05
EML6	229656_s_at	-0.631	7.69E-06
AMDHD1	229596_at	-0.629	0.00055
C6orf89	224988_at	-0.623	0.00095
GREM2	240509_s_at	-0.621	1.45E-05
MYO3A	1569186_at	-0.614	1.50E-05
ABCC4	203196_at	-0.614	2.00E-07
DCP2	235258_at	-0.609	0.00012
HOXC6	206858_s_at	-0.605	2.00E-07
ABCC4	1554918_a_at	-0.601	8.93E-05
JAM2	219213_at	-0.600	2.38E-06
