# Supplementary material for: Analysis of MDM2 and MDM4 Single Nucleotide Polymorphisms, mRNA Splicing and Protein Expression in Retinoblastoma
Source: PLoS One. 2012 Aug 20;7(8):e42739. doi: 10.1371/journal.pone.0042739 (PMC3423419; doi:10.1371/journal.pone.0042739)
Supplement: Materials and Methods S1 — Supplemental materials and methods and supporting references. (PDF) [file pone.0042739.s010.pdf]

Supplemental Materials and Methods

SNP6 Array

Details for the SNP6 arrays have been previously described [1]. The SNP6 array data were deposited in the dbGaP database (phs000352.v1.p1).

SNP Genotype Correlation Analysis

Genotypes for SNP7, SNP309, and SNP34091 were analyzed for correlation with cDNA expression (see cDNA microarray expression). The Kruskal-Wallis test was used for three-group comparisons and the Wilcoxon rank-sum test was used for two-group comparisons.

GST-fusion Constructs and Protein Purification

Details for cloning and purifying recombinant GST-hMDMX (a.a. 1-188) and GST-hMDM2 (a.a. 1-185) protein have been previously described [2].

MDM4 and MDM2 Protein Quantification

Protein lysate (30μg) from cell lines, fetal retina, and orthotopic xenografts were run on an SDS-PAGE Tris-glycine gradient gel (4-15%) alongside 2-fold serial dilutions of purified recombinant GST-MDM2 or GST-MDM4 (to generate standard curve). They were analyzed by immunoblot using primary antibody MDM2 SMP-14 1:200 (Santa Cruz Biotechnology sc-965) or hMDMX/MDM4 1:500 (Bethyl A300-287A-1) followed by IR Dye-labeled secondary antibodies

(anti-rabbit IgG IRDye 680, LI-COR 926-32223 and anti-mouse IgG IRDye 800, LI-COR 926-32210). Please see materials and methods for further immunoblotting procedure. Signal intensity for each band was detected with the Odyssey infrared imager (LI-COR) at 680 and 800 nm. Integrated intensity (counts) was calculated using the Odyssey Imaging software. To generate the standard curve, the integrated intensity for GST-MDM2 and GST-MDM4 proteins was separately plotted per nanograms of total protein loaded. The linear trend line equation was determined for each standard curve. The integrated intensity measured for MDM2 and MDM4 proteins in the cell lines, fetal retina, and orthotopic xenograft tumors was normalized to relative GAPDH levels (relative to GAPDH in U2OS cell line). Next, the normalized integrated intensity values were used to calculate the amount of protein based on the linear trend line equation calculated for each standard curve.

Supplemental References:

1. Zhang J, Benavente CA, McEvoy J, Flores-Otero J, Ding L, et al. (2012) A novel retinoblastoma therapy from genomic and epigenetic analyses. Nature 481: 329-334.
2. Reed D, Shen Y, Shelat AA, Arnold LA, Ferreira AM, et al. (2010) Identification and characterization of the first small molecule inhibitor of MDMX. The Journal of biological chemistry 285: 10786-10796.
